# Estradiol-induced inhibition of endoplasmic reticulum stress normalizes splenic CD4 + T lymphocytes following hemorrhagic shock

**DOI:** 10.1038/s41598-021-87159-1

**Published:** 2021-04-05

**Authors:** Peng Wang, Li-Na Jiang, Chen Wang, Ying Li, Meng Yin, Hui-Bo Du, Hong Zhang, Ze-Hua Fan, Yan-Xu Liu, Meng Zhao, An-Ling Kang, Ding-Ya Feng, Shu-Guang Li, Chun-Yu Niu, Zi-Gang Zhao

**Affiliations:** 1 Institute of Microcirculation, Hebei North University, Diamond South Road 11, Zhangjiakou, Hebei 075000 People’s Republic of China; 2 Pathophysiology Experimental Teaching Center of Basic Medical College, Hebei North University, Zhangjiakou, People’s Republic of China; 3 Department of Gastrointestinal Oncological Surgery, the First Affiliated Hospital of Hebei North University, Zhangjiakou, People’s Republic of China; 4 Basic Medical College, Hebei Medical University, Zhongshan East Road 361, Shijiazhuang, Hebei 075000 People’s Republic of China; 5 Key Laboratory of Critical Disease Mechanism and Intervention in Hebei Province, Shijiazhuang and Zhangjiakou, People’s Republic of China

**Keywords:** Physiology, Medical research

## Abstract

The aim is to investigate that 17β-estradiol (E2)/estrogen receptors (ERs) activation normalizes splenic CD4 + T lymphocytes proliferation and cytokine production through inhibition of endoplasmic reticulum stress (ERS) following hemorrhage. The results showed that hemorrhagic shock (hemorrhage through femoral artery, 38–42 mmHg for 90 min followed by resuscitation of 30 min and subsequent observation period of 180 min) decreased the CD4^+^ T lymphocytes proliferation and cytokine production after isolation and incubation with Concanavalin A (5 μg/mL) for 48 h, induced the splenic injury with evidences of missed contours of the white pulp, irregular cellular structure, and typical inflammatory cell infiltration, upregulated the expressions of ERS biomarkers 78 kDa glucose-regulated protein (GRP78) and activating transcription factor 6 (ATF6). Either E2, ER-α agonist propyl pyrazole triol (PPT) or ERS inhibitor 4-Phenylbutyric acid administration normalized these parameters, while ER-β agonist diarylpropionitrile administration had no effect. In contrast, administrations of either ERs antagonist ICI 182,780 or G15 abolished the salutary effects of E2. Likewise, ERS inducer tunicamycin induced an adverse effect similarly to that of hemorrhagic shock in sham rats, and aggravated shock-induced effects, also abolished the beneficial effects of E2 and PPT, respectively. Together, the data suggest that E2 produces salutary effects on CD4^+^ T lymphocytes function, and these effects are mediated by ER-α and GPR30, but not ER-β, and associated with the attenuation of hemorrhagic shock-induced ERS.

## Introduction

According to current data, death from hemorrhage represents a worldwide problem, with an estimated 1.9 million deaths and 1.5 million of which result from physical trauma in each year^1–3^. Several studies have shown that trauma–hemorrhage induces inhibition of immune function, which reduces the body's ability to resist infection and becomes a key link in the development of hemorrhage to systemic infection^4,5^. Further, cellular immunity dysfunction mediated by splenocytes, such as CD4^+^ T lymphocytes, is considered an important immunological basis of hemorrhagic shock-induced systemic inflammation^6,7^. The previous study showed that hemorrhagic shock induced the damage and dysfunction of splenic CD4^+^ T lymphocytes^8^. Previous studies have revealed that there is gender dimorphism in terms of responses to trauma, shock, and sepsis^9^, and suggested that 17β-estradiol (E2) acts on estrogen receptor-alpha (ERα) to normalize immune function of splenic T lymphocytes or Peyer's patch T cell after trauma-hemorrhage^10–12^. In general, classic estrogen receptors (ERs) are comprised of two nuclear receptors, ERα and ERβ. Recent studies showed that estrogen also have rapid, non-genomic effects which are not mediated by nuclear receptors, but by G protein-coupled receptor 30 (GPR30)^13^. GPR30, is a type of transmembrane estrogen receptor and is also found expression in the endoplasmic reticulum^14^. However, whether ERβ and GPR30 are involved in the beneficial effects of E2 on splenic CD4^+^ T lymphocytes following hemorrhagic shock remains unknown.

Endoplasmic reticulum stress (ERS) is the accumulation of unfolded or misfolded proteins in the lumen of the endoplasmic reticulum, which can be induced by a variety of physiological or pathological conditions. Over-activation of ERS disrupts the physiological function of the endoplasmic reticulum^15^. Extensive studies have demonstrated the cardiac and hepatic dysfunctions were associated with the occurrence of ERS following hemorrhagic shock^16–20^. Although salutary effects of E2 have been reported in male rats following trauma-hemorrhage, it remains unclear whether the salutary effects of E2 on the function of splenic CD4^+^ T lymphocytes following hemorrhage shock are via attenuation of ERS. Therefore, we hypothesized that E2 normalizes the function of splenic CD4^+^ T lymphocytes through inhibition of hemorrhagic shock-induced ERS, which is mediated by activation of ERs. To test it, the present study determined the splenic CD4^+^ T lymphocytes proliferation and cytokine production and splenic histopathology and ERS biomarkers expressions following trauma hemorrhage, and investigated the regulatory effects of E2, the agonist or antagonist of ERs, and the inhibitor or inducer of ERS on these hemorrhagic shock-induced adverse effects.

## Results

### Proliferation of splenic CD4^+^ T lymphocytes

**Figure 1:**
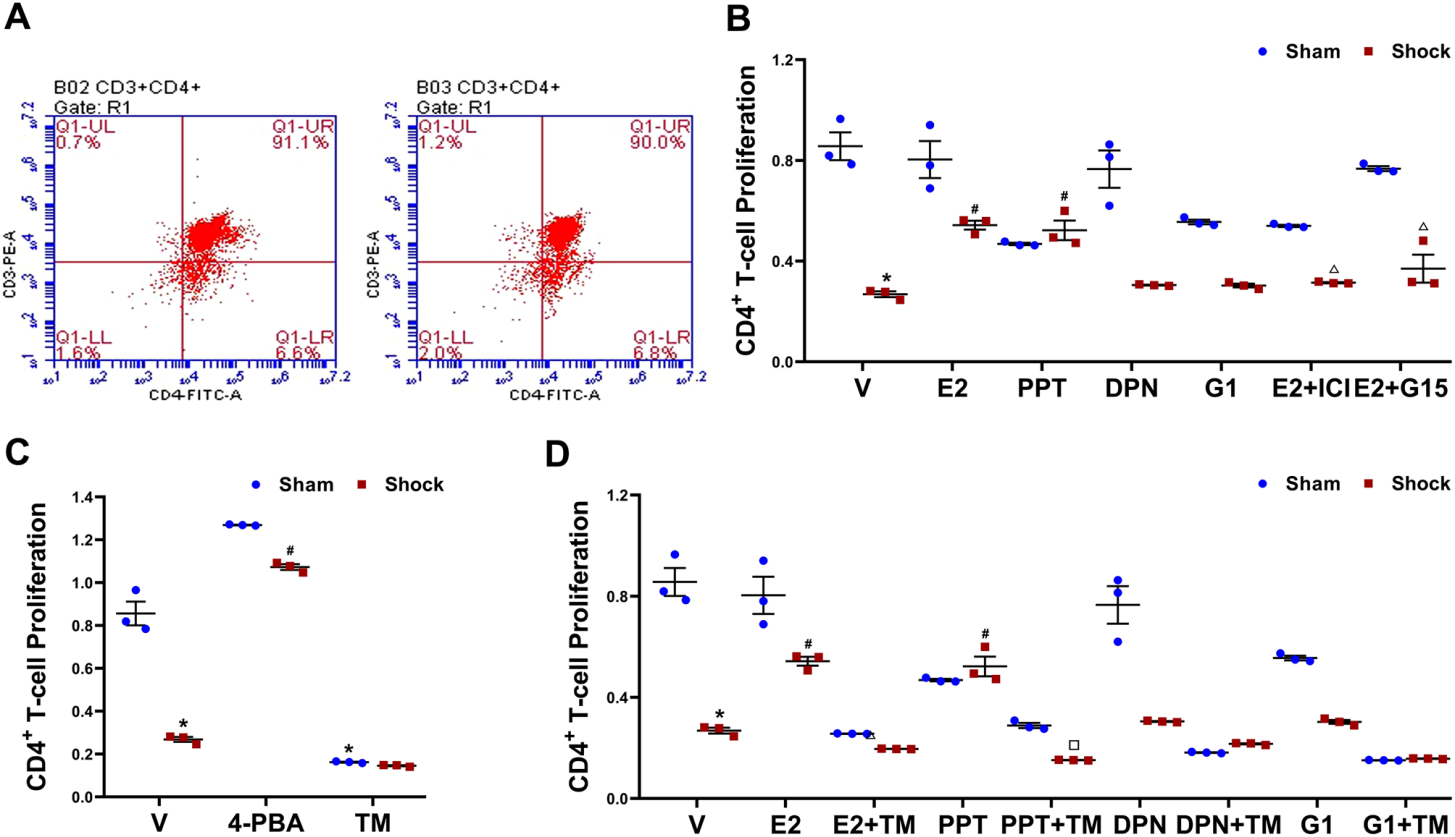
Proliferation of splenic CD4^+^ T lymphocytes isolated from the rats. The animals were sacrificed at 3 h after resuscitation or each time and treated with vehicle (V), 17-estradiol (E2), propyl pyrazole triol (PPT), diarylpropionitrile (DPN), G-1, E2 and ICI 182,780 (E2 + ICI), E2 + G15, 4-Phenylbutyric acid (4-PBA), tunicamycin (TM), E2 + TM, PPT + TM, DPN + TM, G-1 + TM, respectively. The CD4^+^ T lymphocytes were harvested from spleen in rats with the method of immunomagnetic beads separation techniques, flow cytometry analysis demonstrated that cells contained > 90% CD4^+^ T lymphocytes (**A**). The splenic CD4^+^ T lymphocytes (8 × 10^5^ cells/ml) were stimulated with ConA (5 μg/ml) for 48 h and incubated with CCK-8 for 4 h, the proliferation was determined with technical replication of three samples from each splenic tissue, and the proliferative capacity was represented using the optical density measured by the SpectraMax M3 plate reader. Values are mean ± SE of 3 animals in each group (**B**–**D**). B indicated that estrogen receptors (ERs) were involved the role of E2 enhancing the proliferation of splenic CD4^+^ T lymphocytes isolated from the hemorrhagic shocked rats. C indicated that the role of hemorrhagic shock decreasing the proliferation of splenic CD4^+^ T lymphocytes was through the excessive endoplasmic reticulum stress (ERS). D indicated that the beneficial effect of E2 on the proliferation of splenic CD4^+^ T lymphocytes was related to the ERs-dependent inhibition of ERS following hemorrhagic shock. *p < 0.05 vs. the sham + vehicle group, #p < 0.05 vs. the shock + vehicle group, △p < 0.05 vs. the shock + E2 group, □p < 0.05 vs. the shock + PPT group.

The animals were subjected to hemorrhagic shock or sham shock as described previously^21^, and the spleens were collected for the CD4^+^ T lymphocytes isolation at three hours after end of fluid resuscitation or treatments. Flow cytometric analysis demonstrated that obtained cells contained above 90% positive for anti-rat CD3 and anti-rat CD4 (Fig. 1A).

After incubation with Concanavalin A (ConA) for 48 h, the proliferative capacity of CD4^+^ T lymphocytes obtained from hemorrhagic shocked rats that received vehicle at the beginning of resuscitation was significantly decreased compared with that of sham-operated rats receiving vehicle in the presence of ConA (Fig. 1B). Administration of E2 or ERα agonist propyl pyrazole triol (PPT) attenuated the suppressed proliferative capacity of splenic CD4^+^ T lymphocytes under hemorrhagic shock conditions, but there were not statistical changes following ERβ agonist diarylpropionitrile (DPN) or GPR30 agonist G1 treatments (Fig. 1B). In contrast, administrations of ER antagonist ICI 182,780 or GPR30 antagonist G15 abolished the effect of E2 on splenic CD4^+^ T lymphocytes proliferation capacity (Fig. 1B).

The treatment of ERS inhibitor 4-Phenylbutyric acid (4-PBA) significantly inhibited the hemorrhagic shock-induced suppression of CD4^+^ T lymphocytes proliferation. In contrast, ERS inducer tunicamycin (TM) administration reduced the proliferative capacity of CD4^+^ T lymphocytes harvested from the sham rats, and further aggravated the adverse effect of hemorrhagic shock inhibiting CD4^+^ T lymphocytes proliferation to a certain degree, but no statistical difference (P = 0.070) (Fig. 1C). Furthermore, the TM administration abolished the favorable effects of E2 or PPT on proliferation capacity of splenic CD4^+^ T lymphocytes isolated from hemorrhagic shocked rats (Fig. 1D).

### Cytokine production of splenic CD4^+^ T lymphocytes

**Figure 2:**
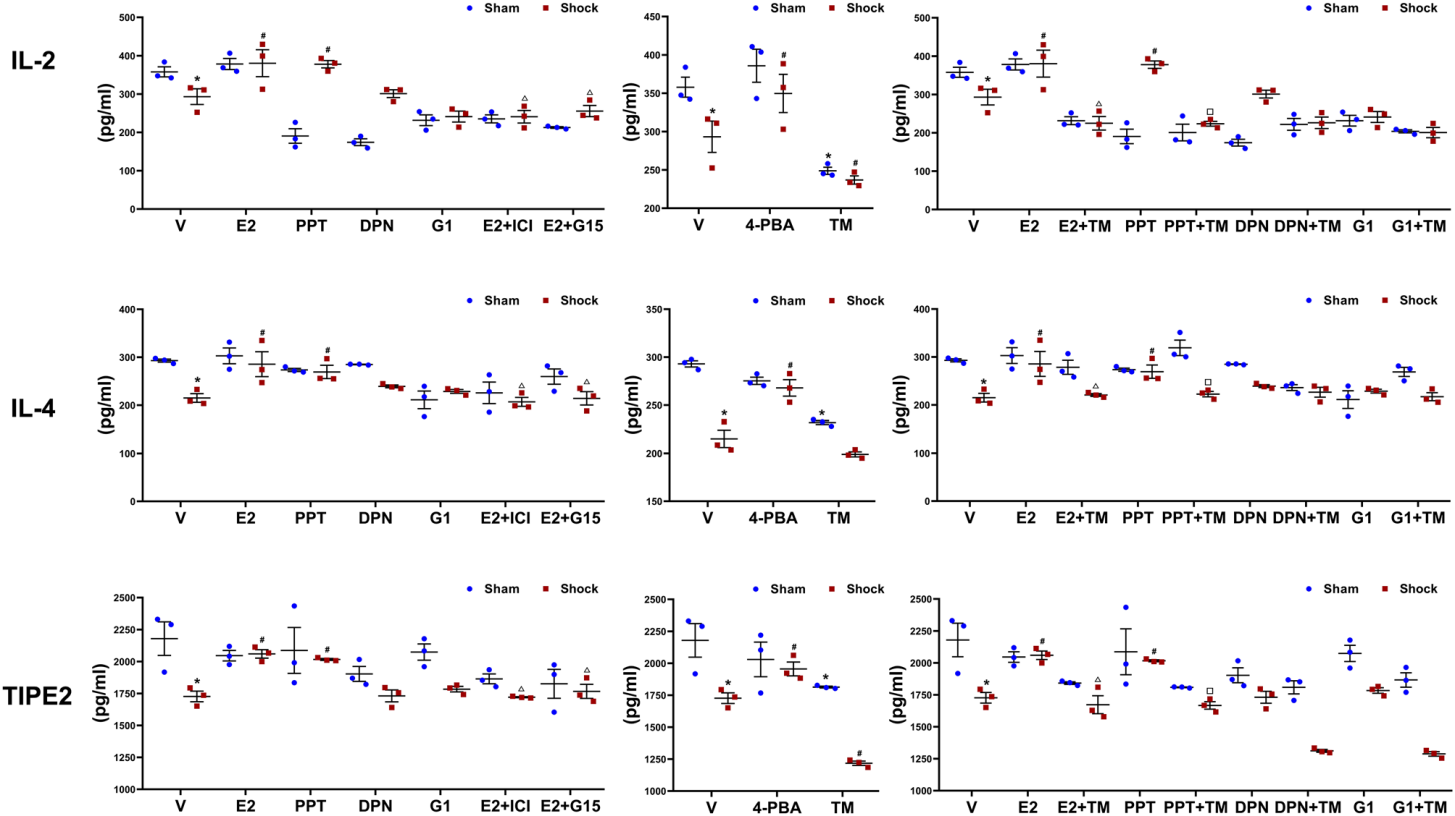
Cytokine production of splenic CD4^+^ T lymphocytes isolated from the rats. The CD4^+^ T lymphocytes were harvested from spleen in rats at 3 h after resuscitation or each time and treated with vehicle (V), 17-estradiol (E2), propyl pyrazole triol (PPT), diarylpropionitrile (DPN), G-1, E2 and ICI 182,780 (E2 + ICI), E2 + G15, 4-Phenylbutyric acid (4-PBA), tunicamycin (TM), E2 + TM, PPT + TM, DPN + TM, G-1 + TM, respectively. Subsequently, the CD4^+^ T lymphocytes were cultured in 96-well plates and stimulated with ConA (5 μg/ml) for 48 h, Cytokine levels of interleukin (IL)-2, IL-4, and tumor necrosis factor-α-induced protein 8 like 2 (TIPE2) in culture supernatants were determined by ELISA. Data are mean ± SE of 3 animals in each group. *p < 0.05 vs. the sham + vehicle group, #p < 0.05 vs. the shock + vehicle group, Δp < 0.05 vs. the shock + E2 group, □p < 0.05 vs. the shock + PPT group.

The CD4^+^ T lymphocytes were cultured in 96-well culture plates with ConA (5 μg/mL) for 48 h, and the cell culture supernatants were collected for the detection of interleukin (IL)-2, IL-4, and tumor necrosis factor-α-induced protein 8 like 2 (TIPE2). The results showed there were no significant differences in the IL-2, IL-4, and TIPE2 productions of splenic CD4^+^ T lymphocytes without stimulation between sham and hemorrhagic shock (data not shown). The contents of IL-2, IL-4 and TIPE2 produced by splenic CD4^+^ T lymphocytes were decreased following trauma-hemorrhage, which were normalized by administrations of E2 or PPT, but not DPN or G1. Furthermore, administrations of either ICI 182,780 or G15 abolished the effects of E2 on splenic CD4^+^ T lymphocytes production of these cytokines (Fig. 2).

In addition, 4-PBA significantly inhibited the cytokine productions of CD4^+^ T lymphocytes isolated from hemorrhagic shocked rats. TM reduced the cytokine productions of CD4^+^ T lymphocytes isolated from the sham rats and further aggravated the adverse effects of hemorrhagic shock. TM also counteracted the effects of E2 or PPT on the cytokines production of CD4^+^ T lymphocytes from rats with hemorrhagic shock (Fig. 2).

### Histopathology of spleen

**Figure 3:**
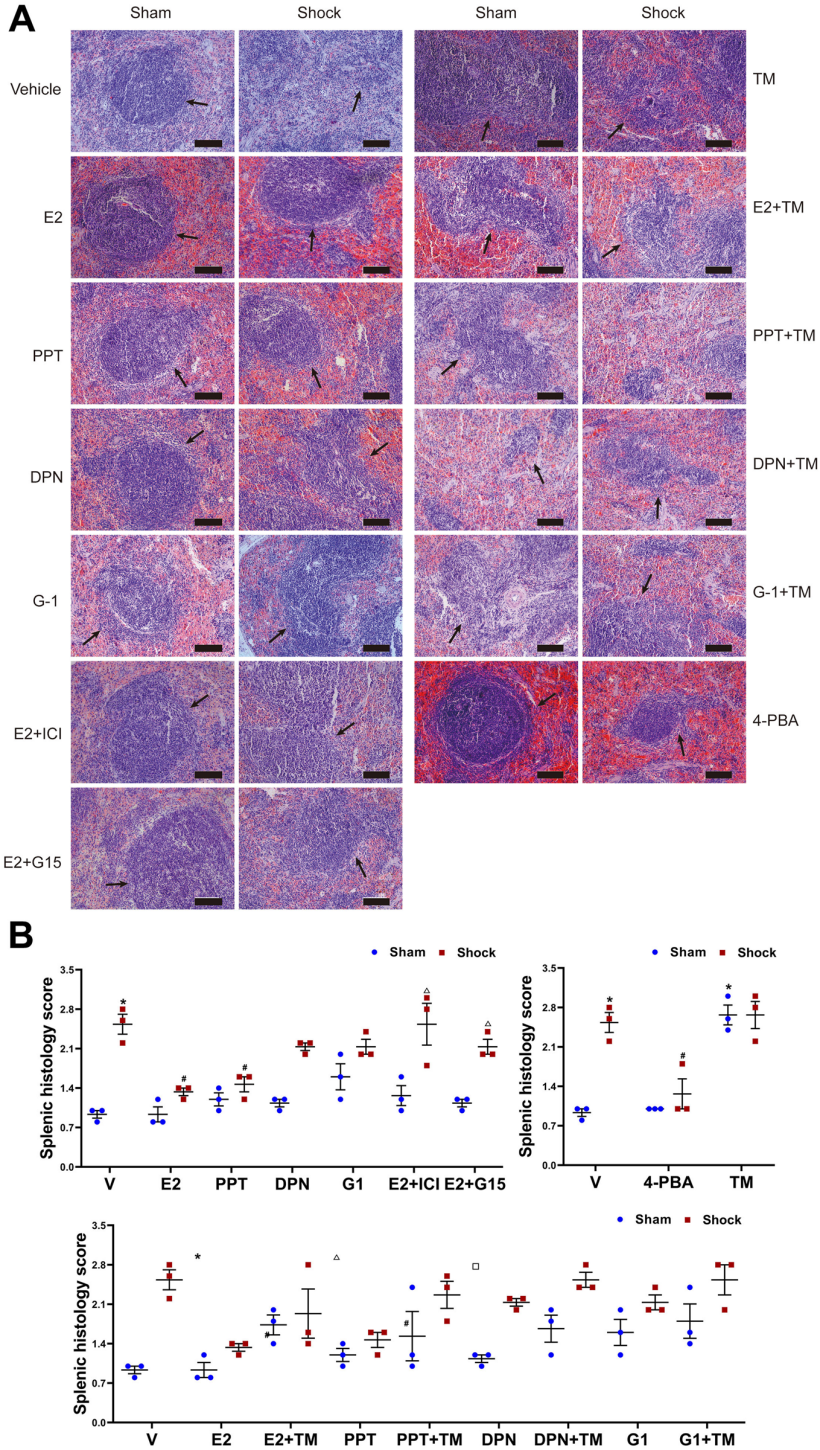
Histological examination of spleen in rats following hemorrhagic shock. The animals were sacrificed at 3 h after resuscitation or each time and treated with vehicle, 17-estradiol (E2), propyl pyrazole triol (PPT), diarylpropionitrile (DPN), G-1, E2 and ICI 182,780 (E2 + ICI), E2 + G15, 4-Phenylbutyric acid (4-PBA), tunicamycin (TM), E2 + TM, PPT + TM, DPN + TM, G-1 + TM, respectively. (**A**) Representative sections of hematoxylin and eosin staining for three animals per group viewed under 20 × objective lens. Arrows indicates the structural change of splenic white pulp from the red pulp regions for the evaluation of splenic structural injury. Scale bar: 100 μm. (**B**) Splenic histology score. Data are mean ± SE of 3 animals in each group. *p < 0.05 vs. the sham + vehicle group, #p < 0.05 vs. the shock + vehicle group, △p < 0.05 vs. the shock + E2 group, □p < 0.05 vs. the shock + PPT group.

Results of histopathological evaluation showed that there were nearly normal constructions with evidences of distinct marginal zone of white pulp and red pulp in the spleen section from the sham rats. In contrast, there was splenic injury with characterizations of missed contours of the white pulp, irregular cellular morphology in that of the shock group. However, ER-α agonist PPT, E2 or 4-PBA administrations following trauma-hemorrhage normalized the contours of the white pulp and significantly decreased the splenic histology score. However, ER-β agonist DPN and GPR30 agonist G1 administrations had no effects on these characterizations. Administration of E2 + ICI 182,780, E2 + G15, E2 + TM or PPT + TM prevented the effects of E2 or PPT on spleen tissues, and observably enhanced the splenic histology score. In addition, TM administration induced spleen tissue damage in the sham rats (Fig. 3).

### Expressions of ERS markers in spleen

**Figure 4:**
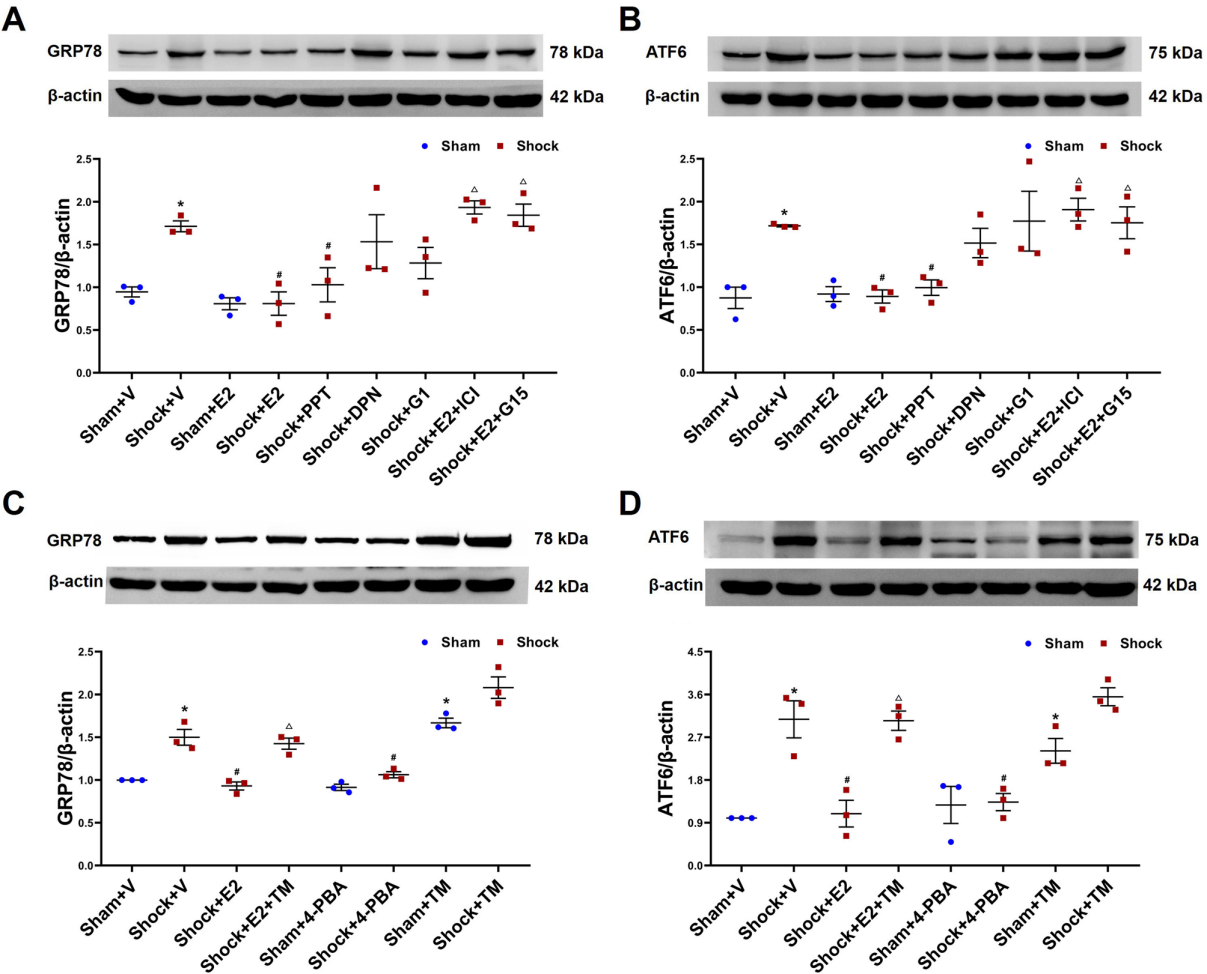
Expressions of GRP78 and ATF6 in spleen. The spleens were harvested from the rats at 3 h after resuscitation or each time and treated with vehicle (V), 17-estradiol (E2), propyl pyrazole triol (PPT), diarylpropionitrile (DPN), G-1, E2 and ICI 182,780 (E2 + ICI), E2 + G15, 4-Phenylbutyric acid (4-PBA), tunicamycin (TM), E2 + TM, respectively. Subsequently, the proteins were extracted and the GRP78 and ATF6 expression were analyzed with the method of Western blotting. GRP78 and ATF6 blots obtained from 3 rats were analyzed using densitometry, and densitometric values were normalized to β-actin and are shown as means ± SD. The figures showed the representative cropped images of western blot analysis of GRP78 and ATF6. Original blots are presented in Supplementary Fig. S1 and Fig. S2. *p < 0.05 vs. the sham + vehicle group, #p < 0.05 vs. the shock + vehicle group, △p < 0.05 vs. the shock + E2 group.

Results from western blotting showed that hemorrhagic shock significantly upregulated the expressions of the ERS protein markers 78 kDa glucose-regulated protein (GRP78) and activating transcription factor 6 (ATF6) when compared to the sham group. Treatments with E2, PPT or 4-PBA at the beginning of resuscitation ameliorated the overexpression of GRP78 and ATF6 under the shock conditions. Meanwhile, ERS inducer TM significantly resulted in increases in the expressions of GRP78 and ATF6 in the sham group, but not E2 or 4-PBA. Furthermore, administrations of ICI 182,780, G15, or TM abolished the effect of E2 on expressions of GRP78 and ATF6 in the shock group (Fig. 4).

## Discussion

The current study demonstrated that either E2/ER activation or inhibition of ERS alleviated hemorrhagic shock-induced decreases in proliferation and cytokine production of the CD4^+^ T lymphocytes, spleen injury and increase in expressions of GRP78 and ATF6. In contrast, blockade of ERα or GPR30, but not ERβ, abolished the salutary effects of E2. Moreover, activation of ERS also abolished the beneficial effects of E2/ER activation. These findings suggest that female sex hormone acts on its receptors to produce salutary effects on hemorrhagic shock-induced CD4^+^ T lymphocytes function through attenuation of ERS.

The evidence indicates that the proliferation of CD4^+^ T lymphocytes plays a major role in immune responses^22^, and low proliferative response of CD4^+^ T lymphocytes is associated with trauma-induced immunosuppression^23^. In general, the levels of production and release of T-helper cell type 1 (Th1) cytokine IL-2, and Th2 cytokine IL-4 were specific embodiment of CD4^+^ T lymphocytes function. Meanwhile, TIPE2 is required for maintaining immune homeostasis^24^. It has been shown that splenocytes proliferation and cytokine production was reduced in males following trauma-hemorrhage^25,26^. However, under same condition, such inhibition of splenocytes function was not observed in proestrus female animals^26^. It follows that there are sex differences in splenocytes proliferation and cytokine production in shock condition^27^. Previous studies^12,28^ have showed that E2 treatment restores the splenocyte proliferation and interferon-gamma (IFN-γ), IL-2, and IL-3 release after trauma-hemorrhage in mice, PPT or E2 administration abolishes hemorrhagic shock-induced decreased production of IL-2 and IFN-γ in isolated T-cell from spleen. In this study, we determined the proliferation and cytokines of IL-2, IL-4, and TIPE-2 in culture supernatants of isolated CD4^+^ T lymphocytes stimulated by ConA and the effect of E2 on these processes. In general, during the detection, ConA binding with its receptors on the surface of T cells nonspecifically activates T cells, then cellular metabolism and morphology are altered successively, leading to changes in proliferation, cell enlargement, cytoplasm enlargement, etc. Hence, ConA plays a stimulating effect and the changes of CD4 + T cell proliferation and cytokine production are related to CD4 + T cell itself. Our findings therefore demonstrated that E2 alters the proliferation and function of cytokine production of CD4^+^ T lymphocytes isolated from hemorrhage shocked rats.

It is well accepted that the biological effects of E2 are mediated by the classic estrogen receptors ERα or ERβ^29^, and non-classic estrogen receptors, such as GPR30^30^. One of recent studies showed that the salutary effects of E2 on T lymphocytes functions are mediated predominantly via ERα following trauma-hemorrhage^12^. Further, they reported the E2 salutary effects on T lymphocytes cytokine production are partially mediated via non-genomic pathway^25^. In the present study, we found that ERα agonist, PPT administration was as effective as E2 in normalizing the proliferation and cytokine production and the values were similarly to shams, whereas ERβ agonist DPN and GPR30 agonist G1 administrations had no obvious effect. In contrast, the effects of E2 on CD4^+^ T lymphocytes were abolished by administrations of ER antagonist ICI 182,780 or GPR30 antagonist G15, suggesting that E2 actions are via both ER-α and GPR30 in these cells. In other words, E2 exerts its effects by either binding with cell-membrane receptors to activate non-genetic signal cascades or directly control genetic transcription after binding with nuclear receptors ERα.

Endoplasmic reticulum, a subcellular organelle, is responsible for the facilitation of protein folding and assembly and involved in several other physiological activities. ERS plays an important role in organic dysfunction and injury following hemorrhagic shock and sepsis^20,31^. The spleen is an organ that is important in combining the both innate and adaptive immunity^32^ and the stress of hemorrhagic shock on endoplasmic reticulum in splenic CD4^+^ T lymphocytes has not been investigated. GRP78 carries a C-terminal endoplasmic reticulum localization KDEL sequence reminiscent and an N-terminal signal sequence of an endoplasmic reticulum resident protein, and high expression of GRP78 inhibits protein synthesis and leads to ERS-induced unfolded protein response (UPR)^33^. Therefore, GRP78 is the primary initiator of early ERS/UPR signaling. ATF6 is a 90 kDa endoplasmic reticulum-resident protein and is identified as a natively unstable protein^34^ and has gained the ability to induce the canonical UPR target genes in higher eukaryotes^35^. Thus, in order to observe the effect of E2 on ERS of splenic CD4^+^ T lymphocytes, the present study assessed the expressions of GRP78 and ATF6 in spleen for the evaluation of ERS, as well as splenic histopathology.

The data in this study showed that hemorrhagic shock increased the expressions of GRP78 and ATF6 in spleen, which were abolished by E2 or PPT administration. More importantly, the administrations of ER antagonist ICI 182,780 or GPR30 antagonist G15 increased the expressions of GRP78 and ATF6, thereby abolishing the salutary effects of E2. Meanwhile, the observation of splenic histopathology also demonstrated that hemorrhagic shock induced structural damage in spleen, and the treatments of E2, PPT, ICI 182,780 and G15 played a similar role with the expressions of ERS markers. Thus, these finding suggest the E2 treatment inhibits the splenic ERS through activation of ERα and GPR30.

In order to further clarify the role of ERS in E2 improving the function of CD4^+^ T lymphocytes function following hemorrhagic shock, firstly, the rats subjected to hemorrhagic shock were treated by the ERS inhibitor 4-PBA. This treatment enhanced the proliferation and restored the ability of produce cytokines of CD4^+^ T lymphocytes isolated from hemorrhagic shock rats, alleviated splenic tissue damage, and reduced the expression of ERS markers. The well-established ERS inductor TM acts via inhibition of N-glycosylation and consequently causes an increase in GRP78^36^. Thus, subsequently, the current study investigated the effects of TM on the sham and shock rats. TM could induce a series of injury changes in sham rats, including the decreased proliferation and ability to produce cytokines of CD4^+^ T lymphocytes, the increased expression of ERS markers and the spleen also appeared structural damage in the splenic tissue. TM administration also aggravated the adverse effects of hemorrhagic shock on these indicators in partly. These results demonstrated not only the effect of TM inducing ERS, but also the role of ERS in the dysfunction of CD4 T cells induced by hemorrhagic shock. Finally, we applied TM to treat the hemorrhagic shock rats combined with either E2, PPT, DPN, or G-1. The data showed that TM abolished the beneficial effects of E2, PPT and G-1 on hemorrhagic shock rats. These finding further indicate that the inhibition of ERS is involved in the mechanism by which E2 normalizes splenic CD4^+^ T lymphocytes function through activation of ERα and GPR30.

We collected the cells and extracted the protein for the examination of ERS markers. However, because the protein amount of the sample was too less for Western blotting analysis, therefore, we extracted the protein from splenic tissue for correlation analysis. Since CD4^+^ T lymphocytes are the main components of splenocytes, we believe that the protein expression of ERS markers in splenic tissue can reflect the expression status of CD4^+^ T lymphocytes.

**Figure 5:**
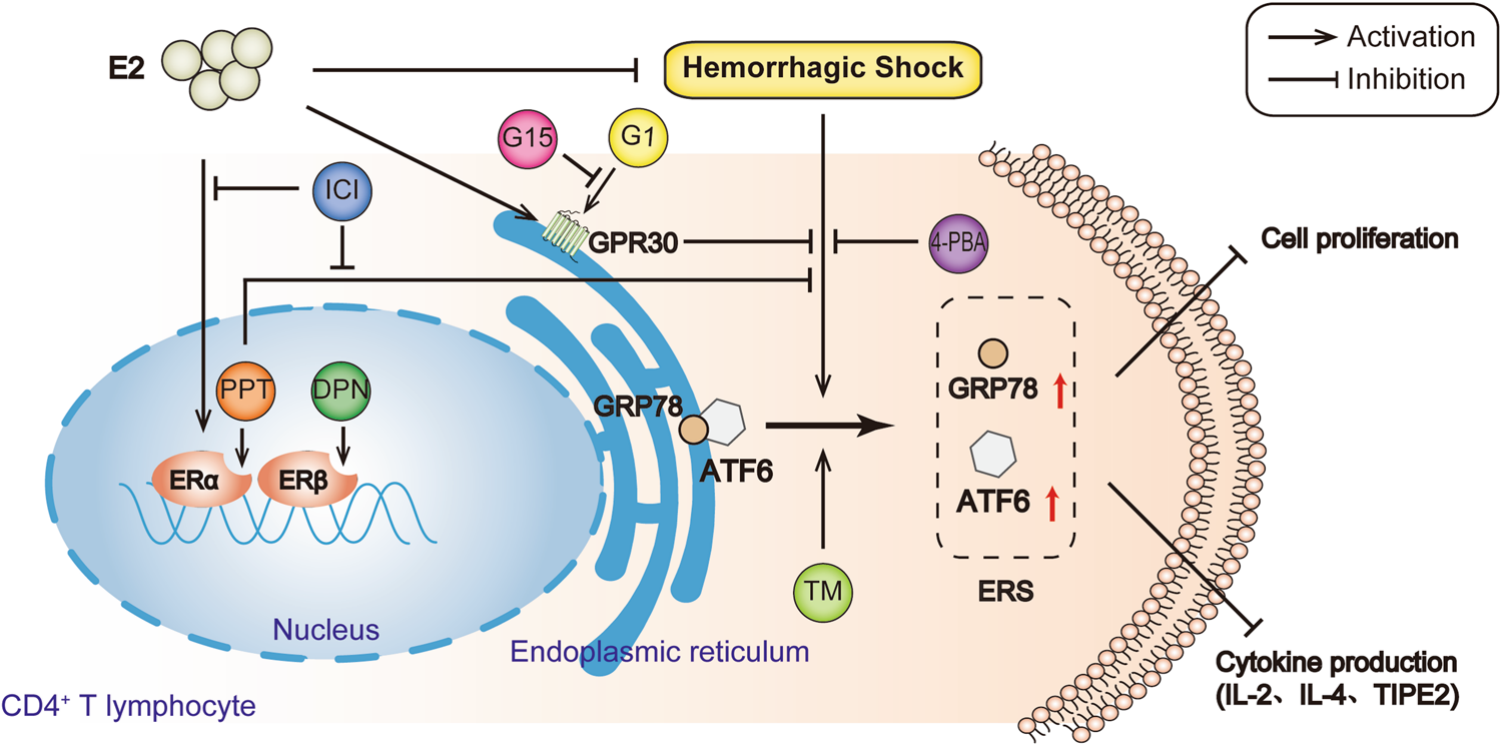
17β-estradiol (E2) inhibits hemorrhagic shock-induced endoplasmic reticulum stress in splenic CD4^+^ T lymphocytes via the estrogen receptor (ER) α and G protein-coupled receptor 30 (GPR30). Hemorrhagic shock induces ERS with evidence of an increase in GRP78 and ATF6 expressions and inhibits the proliferation and cytokines production in splenic CD4^+^ T lymphocytes, which were abolished by E2 treatment through the activations of ERα and GPR30 and 4-PBA administration through the inhibition of ERS. Furthermore, the ERS inducer TM inhibited the favorable roles of E2, PPT and G1 in alleviating hemorrhagic shock-induced ERS in splenic CD4^+^ T lymphocytes.

In conclusion, our results showed that E2 inhibits hemorrhagic shock-induced ERS in splenic CD4^+^ T lymphocytes via actions on the classical pathway ERα or non-genomic pathway GPR30 (Fig. 5). Our finding provides new evidence on E2 improvement of hemorrhagic shock-induced immunosuppression. This study may help to identify new therapeutic targets (ERs) to ameliorate organ damage and immune responses following hemorrhagic shock.

### Limitations

There were several limitations in the current study. The present study observed the salutary effects of E2 on the function of splenic CD4 + T lymphocytes following hemorrhage shock, but did not explore the effect of E2 treatment on lymphoid organ like lymph nodes, peyer’s patches, tonsils and the other immunocytes function including CD8 + T cells, NK cells, and antigen presenting cells (DCs, Macrophages and B cells). Also, this study did not confirm the specificity of E2 treatment to splenic CD4 + T lymphocytes. Therefore, further investigation on the effects of E2 treatment on the lymphoid organ or the other immunocytes is warranted in the future. In addition, IL-17 produced by Th17 cells plays an important role in the intrinsic immune response, primarily through the induction of neutrophil-dominated inflammatory responses, as well as being an important component involved in tissue inflammation^37^. Similarly, CD4 + Foxp3 T cells is involved in the regulation of immune response after hemorrhagic shock^38^. So, the observation of E2 on other types of effectives cells derived from CD4 + T cells, such as Th17 and CD4 + Foxp3 T cells needs to be conducted in future studies. In addition, this study did not observe the splenic structure using staining with appropriate markers, and test the expressions of GRP78 and ATF6 in different cell populations by immunohistochemistry. Combining with the results of E2 treatment on proliferation and cytokine production of isolated CD4 + T cells, these results, to some extent, confirmed the mechanism by which E2 treatment reduces hemorrhagic shock-induced splenic ERS through ERs.

In general, ERS triggers different cellular responses via three ER transmembrane receptors ATF6, double stranded RNA–protein kinase -like ER kinase (PERK), and inositol requiring enzyme/endonuclease 1 (IRE1), which are major three signaling pathways for ERS activation^31,39–41^. Several studies^42,43^ demonstrate that E2 inhibits ceramide–induced ERS in hypothalamic tissue and TM- or dithiothreitol (DTT)–induced ERS in human umbilical vein endothelial cells characterized by decreased expressions of GRP78/ATF6, PERK and IRE1 / X-box binding protein 1 (XBP1), three major branches of the ERS response. Because these data are derived from different models or different pathogenic factors, estrogen plays a universal role in inhibiting ERS, rather than just from a single branch. In this study, we therefore just investigated the changes of GRP78 and ATF6 as the representatives of ERS. Whether the protective effect of E2 is also mediated by inhibiting other ERS branches such as PERK and IRE1/XBP1 needs to be determined in the future.

## Methods

### Animal’s preparation

Adult male (280–320 g) Wistar rats (purchased from the Chinese Academy of Medical Sciences Animal Breeding Center, Beijing, China) were used in this study. All animals were fed under standardized room with an controlled humidity of 40–50% and temperature of 23 ± 2 °C, and 12 h- light–dark cycles (lights on 07∶00 to 19∶00). Basal feed and water were provided, and animals were allowed at least 2 weeks to adapt to the experimental environments. Before the onset of the experiment, the rats were fasted overnight but allowed water ad libitum. All the protocols were performed in adherence to the National Institutes of Health and approved by the Animal Care Committee of Hebei North University (Zhangjiakou, China), and the approval number of the animal experiments was 2017-1-9-05. All surgery was performed under anesthesia, and all rats care followed the “Guide for the Care and Use of Laboratory Animals”. All methods in this study were carried out in compliance with the Animal Research: Reporting of In Vivo Experiments (ARRIVE) guidelines and regulations.

### Hemorrhagic shock model

Rats were lightly induced anesthesia with isoflurane (Hebei Yilin Pharmaceutical Co., LTD., Shijiazhuang, China), and then maintenance of anesthesia with 1% pentobarbital sodium (50 mg/kg, Merck, Germany). Then, rats were restrained in a supine position, and both femoral arteries and the right femoral vein were aseptically cannulated with polyethylene tubing (Smiths Medical International Ltd, Kent, UK) using a minimal dissection technique. Heparin sodium (1 mL/kg, 500U/kg) was injected through the femoral vein for anticoagulation, and blood pressure was measured via one of the femoral arteries using a blood pressure analyzer (ADInstruments, New South Wales, AU), the contralateral femoral artery was connected to an automatic withdrawal-infusion machine (NE-1000, New Era Pump Systems Inc., Farmingdale, NY) for blood withdrawal. Afterwards, an about three cm midline laparotomy (i.e., soft tissue trauma induced) was performed. After a 30-min stabilization period, acute bleeding was carried out rapidly through the other arterial catheter within 10 min to a mean arterial blood pressure (MAP) of 38–42 mmHg, which was maintained at this level for 90 min through pumping or infusing the withdrawal blood as required. Subsequently, the animals received fluid resuscitation with the shed blood volume and equal Ringer’s lactate through the left femoral vein within 30 min. The rats in the sham group underwent the same operation, but neither hemorrhage nor fluid resuscitation.

### Therapeutic protocol

At the onset of the resuscitation, the rats received E2 (2 mg/kg, Merck KGaA, Darmstadt, Germany), PPT (5 μg/kg, Merck KGaA, Darmstadt, Germany), DPN (5 μg/kg, Merck KGaA, Darmstadt, Germany), G-1 (400 μg/kg, ApexBio, Texas, USA), E2 + ICI 182,780 (150 μg/kg, ApexBio, Texas, USA), E2 + G15 (160 μg/kg, ApexBio, Texas, USA), TM (2 mg/kg, ApexBio, Texas, USA), E2 + TM, PPT + TM, DPN + TM, G-1 + TM, ERS inhibitor 4-PBA (20 mg/kg, Merck KGaA, Darmstadt, Germany) or an equal volume of vehicle (20 μL/kg, 100% dimethyl sulfoxide) subcutaneously, respectively. The doses of these reagents are according to the reports from references^44–48^. Among these groups of Shock, Shcok + E2, Shock + PPT, Shock + DPN, Shock + G1, Shock + E2 + ICI, Shock + E2 + G15, the purpose of use of the applied treatment agents were to clarify whether the E2 beneficial effect was achieved through ERs. The aim of 4-PBA and TM administrations were to confirm that ERS involved in hemorrhagic shock-induced CD4 + T cells dysfunction and spleen injury. The protocols of E2, E2 + TM, PPT + TM, DPN + TM, G-1 + TM were to reveal the relationships of E2, ERs, and ERS for the verification of E2 inhibitory effect on ERS through ERs. The sham group was given with the same administration at the same time as a control. Thus, there was twenty-six groups in the current investigation, 6 rats in each group.

### Splenic tissues collection

At three hours after end of fluid resuscitation or treatments, the other experimental protocols were performed for the next observations. In this study, all the rats were humanely sacrificed by cervical dislocation while under deep anesthetic conditions. Subsequently, splenic tissues were obtained aseptically from rats. Three splenic samples in each group were used to CD4^+^ T lymphocytes isolation for the observation of cell proliferation and capacity of producing cytokines in response to ConA in vitro stimulation. While, the other splenic sample was used to splenic histopathological observation and western blotting analysis of the ERS biomarkers.

### CD4^+^ T lymphocytes isolation

Spleens were placed into a 15 mL-centrifuge tube with cold PBS. Then, the spleens were gently ground between syringe piston, and 200 mesh screens was used to produce single cell suspension which was centrifuged at 1500 rpm for 5 min. Then, the isolated splenocytes was resuspended with the phosphate buffer saline (PBS), and the supernatant was discarded. Taking a new centrifuge tube, the splenocytes suspension and the same amount of lymphocyte separation solution were mixed carefully with a pipette, and the miscible liquids were centrifuged at 3000 rpm for 20 min. After centrifugation, the ring-shaped milky white lymphocytes in the middle layer were carefully pipetted into another 15 mL-centrifuge tube, and were then washed three times with PBS.

After cell counting, the centrifuged cells were suspended at 300×g for 10 min, and then the resuspended cells were adjusted in 80 μL of buffer per 10^7^ cells. 20 μL of CD4 MicroBeads (Miltenyi Biotec, Bergisch Gladbach, Germany) were added to the resuspended cells, and were mixed well and incubated for 15 min at 4 °C. Afterwards, the cells were washed with 1 mL of buffer and centrifuged at 300×g for 10 min for removing the supernatant completely.

The centrifuged cells were resuspended with PBS up to a concentration of 10^8^ cells/500 μL. The separation column (Miltenyi Biotec, Bergisch Gladbach, Germany) was prepared by rinsing with 500 μL of buffer in the magnetic field of the magnetic activated cell sorting Separator. And the cell suspension was placed onto the column and then was washed three times with 500 μL of buffer. After removing column from the separator and placing it on centrifuge tube, 1 mL of buffer was pipetted into the column, and the CD4 MicroBeads labeled cells were immediately flushed out from the column, and were washed with PBS.

After centrifugation, the isolated CD4^+^ T lymphocytes were resuspended in RPMI 1640 (Shanghai Lifei Biotechnology Co., Ltd., Shanghai, China) containing 10% heat-inactivated fetal bovine serum and 1% antibiotics to yield a final concentration of 1.6 × 10^6^ cells/mL at 37 °C for the purity identification. The pure CD4^+^ T lymphocytes were used for the next experiment.

### Cell proliferation analysis

The proliferations of CD4^+^ T lymphocytes derived from different groups were determined using the CCK-8 reagent (Applygen, Beijing, China), respectively. Briefly, CD4^+^ T lymphocytes (8 × 10^5^ cells/mL) were plated into a 96-well plate and cultured with ConA (5 μg/mL, Sigma) stimulation at 37 °C, 5% CO_2_ and 95% O_2_ in humidity atmosphere for 48 h. Then, the cells in each well were incubated with CCK-8 for 4 h. Finally, proliferation was determined using the optical density (OD) measured with the M3 microplate reader (Molecular Devices, San Jose, CA) at 450 nm. The proliferation of CD4^+^ T lymphocytes from each splenic tissue was determined with technical replication of three samples and represented with the averaging value.

### Cytokine determination

The levels of IL-2, IL-4, and TIPE2 in culture supernatants were determined using sandwich-enzyme-linked immunosorbent assay method according to the manufacturer’s recommendations (Wuhan ColorfulGene Biological Technology, Wuhan, China). The OD value was measured with a microplate reader at 450 nm. The cytokine level from each supernatant was determined with parallel experiment, and represented with the averaging value.

### Histopathological observation

One part of splenic tissues was washed with cold saline and preserved in 4% paraformaldehyde in the buffered form at 22–24 °C for one week. Afterwards, the spleens were routinely processed and embedded in paraffin. Then sections were cut using rotary microtome (MR2255, Leica, Wetzlar, Germany) and stained with hematoxylin/eosin for histopathological evaluation. Degrees of structural injury in splenic tissues were analyzed by a semi-quantitative scoring system as previously described^49–51^. As Follows: 0, organized splenic white pulp characterized by distinct periarteriolar lymphocyte sheath, germinal center, mantle zone and marginal zone; 1, slightly disorganized splenic white pulp characterized by hyperplastic changes in any region; 2, moderately disorganized splenic white pulp characterized by poorly defined or indistinct areas; and 3, intensely disorganized splenic white pulp characterized by barely distinct from the red pulp regions. Splenic morphological changes were observed using a light microscope (DMI4000B; Leica, Wetzlar, Germany) and images were obtained using an image collection and analysis system (Leica Application Suite 4.0, Leica Microsystems Limited, Switzerland) at magnification of 200 times. Each splenic tissue’ score was determined by the averaging findings from five microscopic fields.

### Western blotting analysis

The remaining part of each splenic tissue was homogenized and lysed, and proteins were extracted with RIPA lysis buffer by using MagNA Lyser System, after which the samples were centrifuged at 12,000×g for 10 min at 4 °C. Protein samples were loaded on 10%–15% SDS-PAGE and transferred to polyvinylidene fluoride (PVDF) membranes using a Mini Trans-Blot Electrophoretic Transfer Cell (Bio-rad, USA). The membranes were blocked in 5% nonfat milk for 1 h and then incubated with the primary antibodies anti-ATF6 (ab203119, Abcam Inc., Cambridge, MA) at 1:1000 and anti-GRP78 (ab21685, Abcam Inc., Cambridge, MA) at 1:1000 at 4 °C overnight, respectively. The membranes were then washed three times with TBS-Tween (TBS-T) and incubated with the secondary antibodies conjugated to horseradish peroxidase (HRP) for 1 h, and washed again with TBS-T. Blots were probed using chemiluminescence detection reagents (Applygen Technologies Inc., Beijing, China) and exposure to ImageQuant (LAS 4000, General Electric Company, Boston, MA). The band intensities of the proteins of interest were qualified using Quantity One v4.6.2 software and normalized by comparison to the intensity of β-actin.

### Statistical analysis

The results of cell proliferation, cytokine production and splenic histology score are presented as means ± standard error (SE), and the result of western blotting is presented as means ± standard deviation (SD). One-way analysis of variance (ANOVA) followed by Tukey test as a post hoc test for multiple comparisons was used to determine the significance of the differences between groups. The differences were considered significant if *P* < 0.05. Statistical analysis was performed using SPSS 16.0 for Windows (SPSS Inc., Chicago, IL).

### Ethics approval and consent to participate

All the protocols were approved by the Animal Care Committee of Hebei North University (Zhangjiakou, China) with the approval number of 2017-1-9-05.

## Supplementary Information

Supplementary Information.

## Data Availability

The data generated for this study are available on request to the corresponding author.
