# Supplementary material for: Estradiol-induced inhibition of endoplasmic reticulum stress normalizes splenic CD4 + T lymphocytes following hemorrhagic shock
Source: Sci Rep. 2021 Apr 5;11:7508. doi: 10.1038/s41598-021-87159-1 (PMC8021564; doi:10.1038/s41598-021-87159-1)
Supplement: Supplementary file 1 — Supplementary Information. [file 41598_2021_87159_MOESM1_ESM.pdf]

Estradiol-induced inhibition of endoplasmic reticulum stress normalizes splenic CD4+ T lymphocytes following hemorrhagic shock

Peng Wang[1,2], Li-Na Jiang[1], Chen Wang[1,2], Ying Li[1,2], Meng Yin[1,2], Hui-Bo Du[1], Hong Zhang[1,2], Ze-Hua Fan[1,2], Yan-Xu Liu[1,2], Meng Zhao[1,2], An-Ling Kang[1,2], Ding-Ya Feng[1,2], Shu-Guang Li[1,3], Chun-Yu Niu[4,5*], Zi-Gang Zhao[1,2,5*]

[1] Institute of Microcirculation, Hebei North University, Zhangjiakou, PR China.

[2] Pathophysiology Experimental Teaching Center of Basic Medical College, Hebei North University, Zhangjiakou, PR China.

[3] Department of Gastrointestinal Oncological Surgery, the First Affiliated Hospital of Hebei North University, Zhangjiakou, PR China.

[4] Basic Medical College, Hebei Medical University, Shijiazhuang, PR China.

[5] Key Laboratory of Critical Disease Mechanism and Intervention in Hebei Province, Shijiazhuang and Zhangjiakou, PR China

Address correspondence to

1. Zi-Gang Zhao, Institute of Microcirculation, Hebei North University, Diamond South Road 11, Zhangjiakou, Hebei 075000, People's Republic of China. Tel: +86-18903132966, +86-313-4029223; E-mail address: zzghyl@126.com.
2. Chun-Yu Niu, Basic Medical College, Hebei Medical University, Zhongshan East Road 361, Shijiazhuang, Hebei 075000, People's Republic of China. Tel: +86-18931318886, +86-311-86266215; E-mail: ncylxf@126.com.

Supplementary Figures

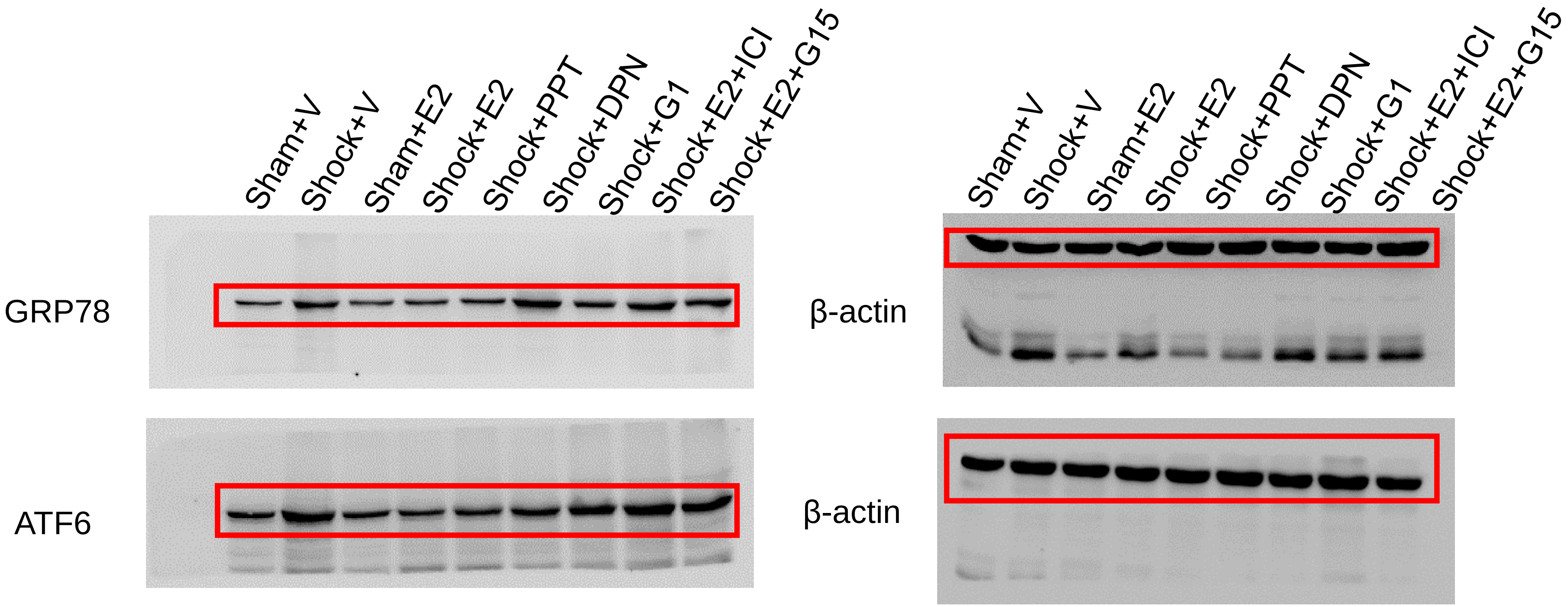

Supplementary Fig. S1 Original blots related to Fig. 4A and Fig. 4B.

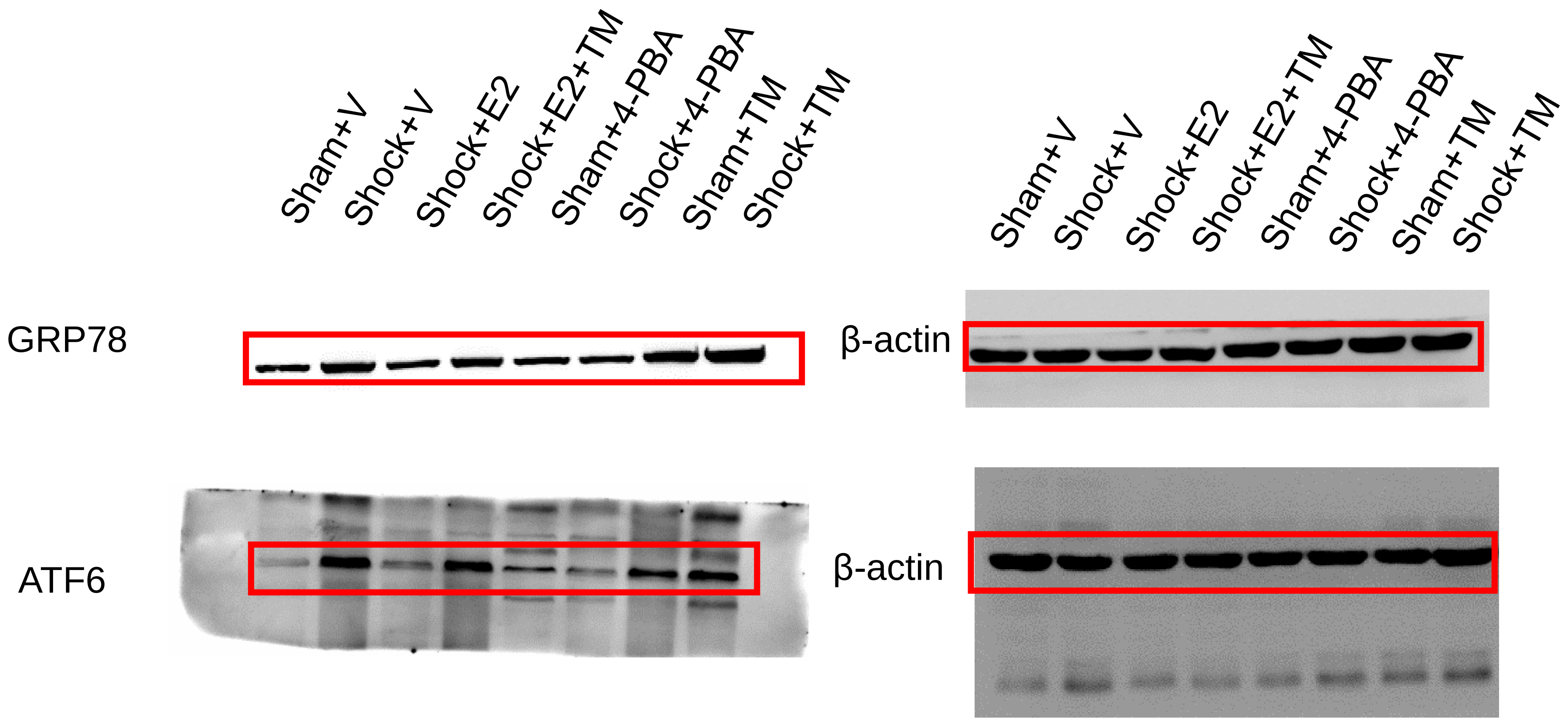

Supplementary Fig. S2 Original blots related to Fig. 4C and Fig. 4D.
